# Intensive care following in-hospital cardiac arrest / periarrest calls—experience from one Scottish hospital

**DOI:** 10.1186/s44158-023-00136-0

**Published:** 2024-01-02

**Authors:** Andrew R. McCallum, Richard Cowan, Kevin D. Rooney, Paul C. McConnell

**Affiliations:** 1 Department of Anaesthesia, Queen Elizabeth University Hospital, 1345 Govan Rd, Glasgow, G51 4TF UK; 2 Department of Anaesthesia and Intensive Care Medicine, Glasgow Royal Infirmary, 84 Castle St, Glasgow, G4 0SF UK; 3 Department of Anaesthesia and Intensive Care Medicine, Royal Alexandra Hospital, Corsebar Rd, Paisley, PA2 9PN UK

**Keywords:** Cardiac arrest, Periarrest, ICU referrals, ICU admission

## Abstract

**Background:**

In-hospital cardiac arrest/periarrest is a recognised trigger for consideration of admission to the intensive care unit (ICU). We aimed to investigate the rates of ICU admission following in-hospital cardiac arrest/periarrest, evaluate the outcomes of such patients and assess whether anticipatory care planning had taken place prior to the adult resuscitation team being called.

**Methods:**

Analysis of all referrals to the ICU page-holder within our district general hospital is between 1st November 2018 and 31st May 2019. From this, the frequency of adult resuscitation team calls was determined. Case notes were then reviewed to determine details of the events, patient outcomes and the use of anticipatory care planning tools on wards.

**Results:**

Of the 506 referrals to the ICU page-holder, 141 (27.9%) were adult resuscitation team calls (114 periarrests and 27 cardiac arrests). Twelve patients were excluded due to health records being unavailable. Admission rates to ICU were low — 17.4% for cardiac arrests (4/23 patients), 5.7% (6/106) following periarrest. The primary reason for not admitting to ICU was patients being “too well” at the time of review (78/129 — 60.5%). Prior to adult resuscitation team call, treatment escalation plans had been completed in 27.9% (36/129) with Do Not Attempt Cardiopulmonary Resuscitation (DNACPR) forms present in 15.5% of cases (20/129). Four cardiac arrest calls were made in the presence of a valid DNACPR form, frequently due to a lack of awareness of the patient’s resuscitation status.

**Conclusions:**

This study highlights the significant workload for the ICU page-holder brought about by adult resuscitation team calls. There is a low admission rate from these calls, and, at the time of resuscitation team call, anticipatory planning is frequently either incomplete or poorly communicated. Addressing these issues requires a collaborative approach between ICU and non-ICU physicians and highlights the need for larger studies to develop scoring systems to aid objective admission decision-making.

## Introduction

Within the UK, the incidence of in-hospital cardiac arrest is estimated at approximately 1.6 per 1000 hospital admissions. Return of spontaneous circulation (ROSC) is estimated to occur in around 45%, with survival to hospital discharge occurring in 18% [1].

Within our hospital, the ICU page-holder forms part of the Adult Resuscitation (AR) team, who urgently attend all calls for immediate assistance on the wards due to either cardiac arrest or ward concern of severe physiological derangement needing urgent treatment to prevent cardiac arrest (“periarrest”). Such calls are often triggers for consideration for admission to the intensive care unit (ICU). This may not be appropriate for all patients, depending on whether admission will likely convey additional benefit [2]. Treatment escalation plans (TEPs), discussed with patients well in advance of such emergencies, are important in guiding these decisions and have been shown to be effective in preventing inappropriate ICU referrals [3].

Evidence from the ICU literature regarding in-hospital cardiac arrest tends to focus on outcomes following ICU admission [4]. There is, however, little published with regard to referrals from such emergency calls. We therefore aimed to investigate the rates of ICU admission following in-hospital cardiac arrest/periarrest, evaluate the outcomes of such patients and assess whether anticipatory care planning had taken place prior to the AR team being called.

## Methods

The Royal Alexandra Hospital is a district general hospital with approximately 760 beds and 7 level three ICU beds. As a measure of our current practice, continual data collection of all referrals made to the ICU page-holder (including cardiac arrest/periarrest calls) runs within our department. As part of this, following all referrals, the ICU page-holder completes a proforma detailing patient demographics, the background of the case and the immediate outcome of the referral. Data are electronically transcribed, from which our analysis was performed. Caldicott Guardian approval was obtained for the dataset and ethical approval was deemed unnecessary by the local research ethics service.

Data presented here covers the period between 1st November 2018 and 31st May 2019. Each AR call was considered a separate entity. Cardiac arrest calls were defined as the initiation of cardiopulmonary resuscitation (CPR) and/or artificial ventilation at the time of referral; all other AR calls were classified as periarrests.

The outcome from the review by the ICU page-holder was initially classified into admission or non-admission to level three care. Those not admitted to ICU were then either considered to be “too well” or “too unwell” to benefit from admission. “Too well” represents those patients whose physiological state, upon review, did not currently require a higher degree of organ support or supervision beyond that available in the patient’s current area of care. “Too unwell” represents those patients where, as a result of their current physiology being deemed unsalvageable and/or their chronic co-morbidities and lack of physiological reserve being assessed as too severe, escalation to multiple organ support in the ICU was assessed to offer no benefit to the patient. The ICU page-holder (usually an anaesthetic trainee or anaesthetic specialty doctor) could, based on their review, determine a patient was “too well” to benefit from ICU. However, where there was any doubt (and in all cases where the page-holder considered the patient “too unwell”), discussion with the on-call ICU consultant was mandatory. Only after this expert discussion could a patient be considered “too unwell” to benefit from ICU. As is our normal practice, all overnight referrals were then further discussed at the morning handover, allowing for a second consultant intensivist opinion.

The health records of identified patients were then retrospectively reviewed, with patients excluded from further analysis in cases where these were unavailable. These records were used to determine mortality and the presence/absence of both TEPs and Do Not Attempt Cardiopulmonary Resuscitation (DNACPR) forms. If a DNACPR form was present, the date of completion was cross-referenced against the time of the AR call. If the call occurred following the completion of a DNACPR form, then medical notes were further evaluated to ascertain the reason for the AR team call. Mortality data are correct as of July 2019. Data are presented as count (%) and median (IQR).

## Results

**Fig. 1:**
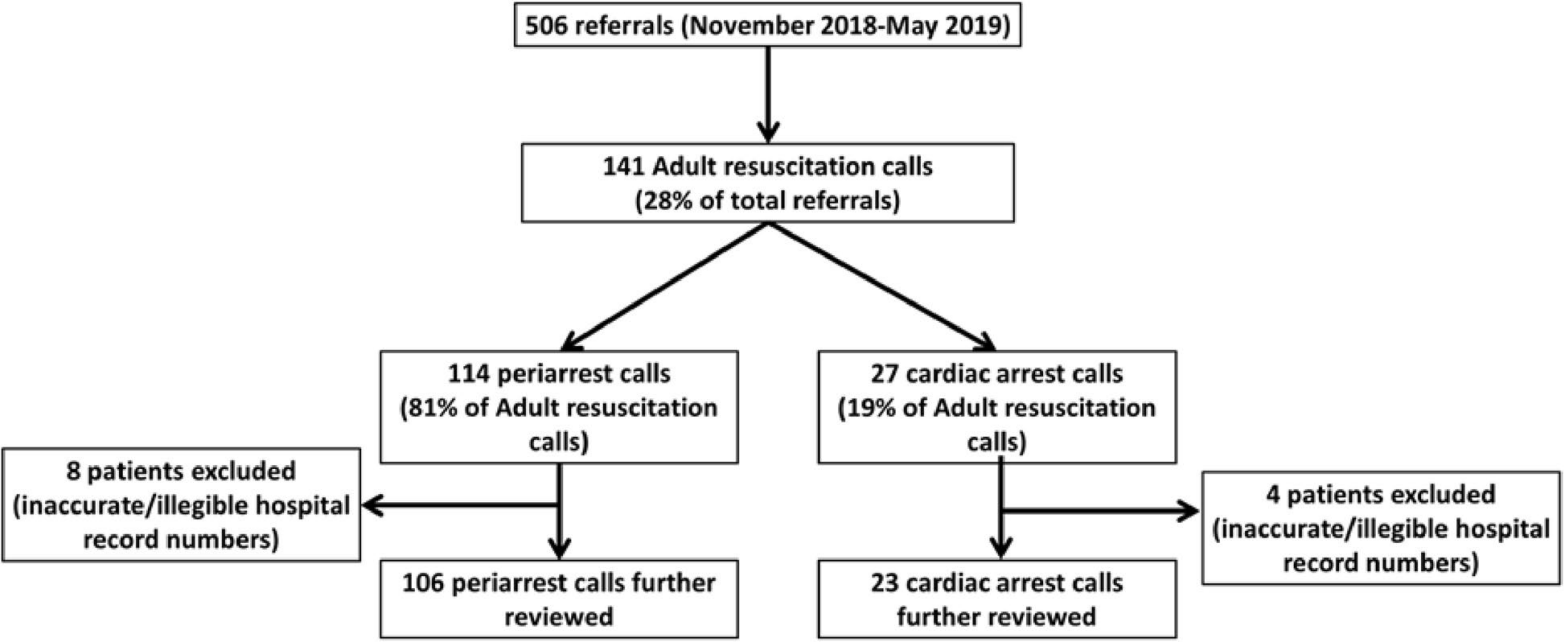
Breakdown of investigated patients

A total of 506 ICU referrals were made over the 7-month study period. This comprised 114 periarrests and 27 arrest calls and represented 27.9% of total ICU referrals over the time period. Accounting for the potential for patients to have multiple AR calls over a single hospital admission, 131 individual patients were attended over this period. The profile of studied AR calls is shown in Fig. 1.

Characteristics of included cases are shown in Table 1. The acute medical receiving unit produced the highest call volume (14/141 calls — 9.9%) followed by the inpatient respiratory ward (12/141 calls, 8.5%). The High Dependency Unit (HDU) and Coronary Care Unit (CCU) each produced around 7% of calls (10/141 and 11/141, respectively).

**Table 1:** Characteristics of adult resuscitation team calls

**Time of day of arrest (count, (%))**	
Morning (06.00–11:59)	37 (28.7%)
Afternoon (12:00–17:59)	37 (28.7%)
Evening (18:00–23:59)	32 (24.8%)
Night (00:00–05:59)	20 (15.5%)
Not recorded	3 (2.3%)
**Grade of ICU page-holder at time (count, (%))**	
Core trainee (CT1-CT2)	74 (57.4%)
Specialist trainee (ST3-ST7)	49 (38.0%)
Specialty doctor or associate specialist (SAS)	6 (4.7%)
**Number of level 3 beds occupied at time call (Median [IQR])**^**a**^	5 [3–6]

^a^Data available in 49 cases. Total of 7 level three beds available within hospital

**Fig. 2:**
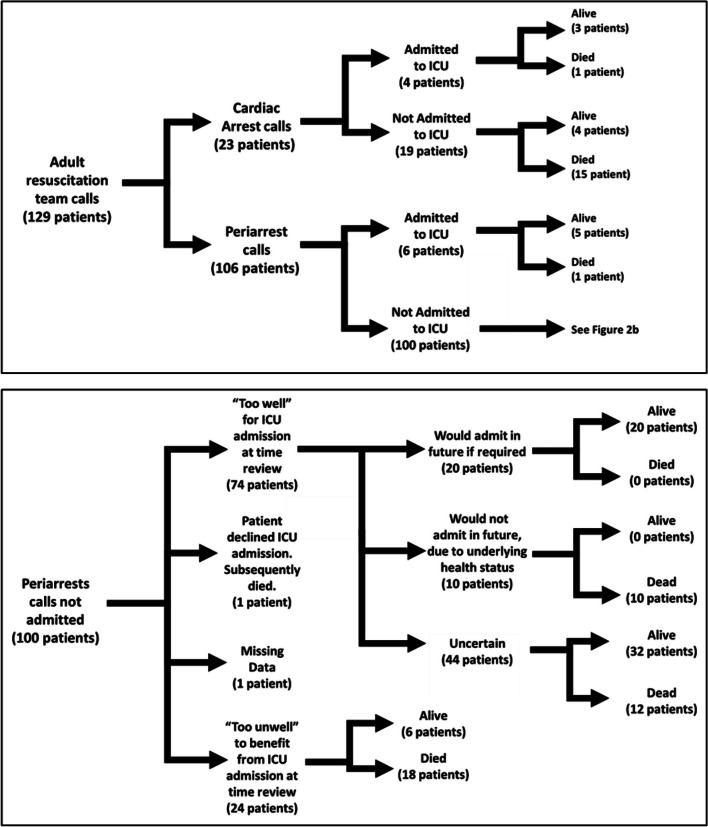
Outcome of periarrest/arrest calls

After applying exclusions, 129 patients were studied further (23 having suffered a cardiac arrest and 106 a periarrest). The overall admission rate to ICU from these calls was 7.7% (10/129). For post-cardiac arrest, the admission rate was 17.4% (4/23 patients); for periarrests, it was 5.7% (6/106). Of the 119 patients not admitted to ICU, the majority (84 patients — 70.6%) were felt to be “too well”, whilst a further 29.4% (35 patients) were felt to be “too unwell” to benefit from ICU admission. Overall in-hospital mortality following a cardiac arrest/periarrest call was 45.0% (58/129). For cardiac arrest patients the mortality rate was 69.6% (16/23 patients), for periarrests, it was 39.6% (42/106). Amongst those admitted to ICU, overall mortality was 20.0% (2/10 patients), with similar figures seen in both cohorts — 25.0% for cardiac arrests (1/4 patients), 16.7% for periarrests (1/6 patients). These results are summarised in Fig. 2a and b.

**Fig. 3:**
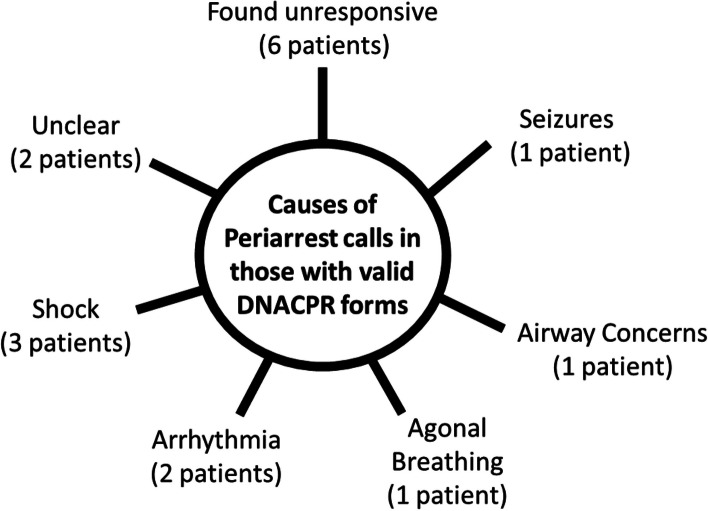
Reasons for periarrest calls being placed for those with a valid DNACPR

Treatment escalation plans had been completed in 36 patients (27.9%) prior to the AR call. DNACPR forms were present in the notes of 20 patients prior to AR call (15.5%). There were four cardiac arrest calls in patients with a valid DNACPR form (17.4%); in three of these cases, a delay in recognising the presence of a valid DNACPR form appears to have occurred, leading to the commencement of cardiopulmonary resuscitation. In the final case, the validity of the form was questioned and CPR commenced in the interim. In all cases, resuscitation was ultimately unsuccessful. Of the 16 patients with valid DNACPR forms for whom a periarrest call was made (15.1%), there were a variety of causes (as shown in Fig. 3).

## Discussion

This study investigated the incidence of AR calls within our district general hospital between November 2018 and May 2019. We found that over a quarter of calls to the ICU referral page were due to arrests and periarrest calls, and that the vast majority of these patients either do not need, or would not benefit from, level three care. There is a frequent lack of anticipatory care planning in these patients. CPR is also commenced in a considerable number of patients with a valid DNACPR form.

The low frequency of admissions to ICU arising from AR calls is an important finding. This is primarily due to patients being “too well” for ICU at the time of review. A median of 5 patients (IQR 3–6) were already admitted to the intensive care unit at the time of AR call during this study. This represents at least a 71.4% level 3 bed occupancy and may be higher, depending on levels of nursing staffing during any one shift. Out-of-hours, the ICU page-holder may be the only doctor onsite covering these patients. The ethics of this situation need balancing: attending AR calls separates the ICU page-holder from critically ill patients already admitted to the ICU. However, attendance of page-holders at AR calls may bring additional decision-making ability and when their specialist skills are required, this need is often immediate. No international standards exist regarding the composition of AR teams and the makeup varies between sites [5]. Resuscitation Council UK standards require resuscitation teams to contain a minimum skill set, rather than comprise of any specific individuals [6]. Our study suggests organisations should examine their local set-ups to determine if the status quo provides the best utilisation of resources.

Early anticipatory planning is a primary driver of the Scottish Patient Safety Programme’s Structured Response to the Deteriorating Patient [7]. This study shows treatment escalation plans are frequently incomplete at the time of cardiac arrest/periarrest. Interestingly, amongst patients deemed “too well” for ICU at time of the AR call, 26.9% (21/78) subsequently had DNACPR forms completed during their admissions. Whilst there are many potential reasons for this, it implies earlier discussions on the appropriateness of various treatments may have been indicated in a number of our patients. To maximise future effectiveness, in cases where there is uncertainty, the treatment escalation planning process should be formalised, with collaborative discussions between ICU and non-ICU physicians, taking into account patient and family input.

Our data also demonstrates that whilst there is a high mortality amongst periarrest patients deemed unlikely to gain benefit from ICU, a considerable proportion (25.0%) of these patients survive. Refusal of ICU admission does not equate to a withdrawal of care, rather that additional critical care interventions are unlikely to alter the ultimate outcome. This is a vital distinction which needs to be communicated effectively to the ward team to ensure optimal patient care.

The findings regarding the prevalence of arrest calls being placed in those with valid DNACPR forms are a concern. Analysis of our data suggests that a common theme is the lack of awareness of a patient’s resuscitation status. Further work at a ward level (and at time of admission) is required to improve the communication of anticipatory care plans amongst all those involved in patient care.

This study is limited by being single centre. The variation in compositions of AR teams, differing set-ups of critical care units and the lack of a national referral recording database makes studying outcomes of ICU referrals at a wider level challenging. The effect of the COVID-19 pandemic on admissions and outcomes from AR team referrals is also unknown.

This study represents a review of our normal practice and demonstrates the challenges non-ICU physicians face in preventing inappropriate ICU referrals. The subjective (although expert) nature of decision-making by ICU physicians regarding level three admission may contribute to some of the uncertainty faced at the ward level. There is a worldwide need to refine and reach agreement on what constitutes a futile ICU referral. Scoring systems exist to predict outcome once admitted to ICU but do not guide decision-making for admission [8]. Similarly, scoring systems (based on pre-arrest factors) exist to predict survival following in-hospital cardiac arrest but are neither widely used nor do they consider the periarrest population [9]. A further larger, multicentre study would potentially allow for the development of a scoring system to aid in objectifying admission decision-making in such acute scenarios. Such a study may also help to objectify the criteria used to define the”too well” and”too unwell” patient groups. Considering the “too well” survivors and “too unwell” non-survivors could also allow for delineation of the differences between these groups, potentially providing valuable prognostic information for both ICU and non-ICU physicians.

In conclusion, we demonstrate that adult resuscitation team calls contribute significantly to the referral workload of the ICU page-holder, although admission rates from such calls are low. Whilst the utility of routinely attending all AR calls can be reviewed, this does not address an important underlying problem: namely insufficient escalation planning and poor ward communication of resuscitation status. Correcting these factors may decrease inappropriate ICU referrals and improve patient care, by ensuring only appropriate and useful interventions are undertaken. These goals are in line with those of a realistic medicine framework [10].

## Data Availability

The datasets used and/or analysed during the current study are available from the corresponding author on reasonable request.
